# LET-SE2-VINS: A Hybrid Optical Flow Framework for Robust Visual–Inertial SLAM

**DOI:** 10.3390/s25133837

**Published:** 2025-06-20

**Authors:** Wei Zhao, Hongyang Sun, Songsong Ma, Haitao Wang

**Affiliations:** 1 School of Mechanical and Electrical Engineering, Shenzhen Polytechnic University, Shenzhen 518055, China; zhaowei@szpu.edu.cn (W.Z.); 202217506@mail.sdu.edu.cn (H.S.); wanghaitao@szpu.edu.cn (H.W.); 2 School of Mechanical, Electronic & Information Engineering, Shandong University, Weihai 264209, China

**Keywords:** hybrid optical flow method, VI-SLAM, deep learning, key point detection, enhance localization accuracy

## Abstract

This paper presents SE2-LET-VINS, an enhanced Visual–Inertial Simultaneous Localization and Mapping (VI-SLAM) system built upon the classic Visual–Inertial Navigation System for Monocular Cameras (VINS-Mono) framework, designed to improve localization accuracy and robustness in complex environments. By integrating Lightweight Neural Network (LET-NET) for high-quality feature extraction and Special Euclidean Group in 2D (SE2) optical flow tracking, the system achieves superior performance in challenging scenarios such as low lighting and rapid motion. The proposed method processes Inertial Measurement Unit (IMU) data and camera data, utilizing pre-integration and RANdom SAmple Consensus (RANSAC) for precise feature matching. Experimental results on the European Robotics Challenges (EuRoc) dataset demonstrate that the proposed hybrid method improves localization accuracy by up to 43.89% compared to the classic VINS-Mono model in sequences with loop closure detection. In no-loop scenarios, the method also achieves error reductions of 29.7%, 21.8%, and 24.1% on the MH_04, MH_05, and V2_03 sequences, respectively. Trajectory visualization and Gaussian fitting analysis further confirm the system’s good robustness and accuracy. SE2-LET-VINS offers a robust solution for visual–inertial navigation, particularly in demanding environments, and paves the way for future real-time applications and extended capabilities.

## 1. Introduction

SLAM (Simultaneous Localization and Mapping) is a critical technology that enables robots to perform self-localization and map construction simultaneously in unknown environments. As one of the core technologies in robotics, autonomous driving, drones, and augmented reality, it plays a pivotal role in enabling autonomous navigation and environmental perception. By utilizing laser radar, SLAM can accurately measure the distances of objects within the environment, making it highly suitable for constructing precise 2D or 3D geometric maps [1,2,3]. Notably, this technology is independent of ambient lighting conditions, allowing it to operate reliably in both dark and brightly lit environments [4,5].

While laser radar may struggle to effectively detect transparent or reflective objects, visual sensors can address these limitations by leveraging texture and visual information. Visual SLAM (V-SLAM), an important branch of SLAM technology, effectively resolves this issue. Primarily relying on visual sensors for localization and mapping, V-SLAM functions similarly to the human eye, capturing detailed environmental information to construct a model of the surroundings while simultaneously estimating the robot’s movement [6,7].

V-SLAM has undergone significant development over the years. In 2007, Davison et al. introduced MonoSLAM, the first real-time V-SLAM method based on the Extended Kalman Filter (EKF). However, its single-threaded architecture and real-time constraints limited the number of trackable front-end features [8]. To address this limitation, Kameda et al. proposed the Parallel Tracking and Mapping (PTAM) method [9]. PTAM revolutionized the field by employing parallel processing, dividing tracking and map-building tasks into two separate threads, thereby enhancing real-time performance. Additionally, it pioneered the use of nonlinear optimization for computing camera trajectories and global map information. Building on these advancements, Mur-Artal et al. developed the Oriented FAST and Rotated BRIEF SLAM 2 (ORB-SLAM2) method, which integrated monocular, binocular, and Red Green Blue Depth (RGB-D) modes, significantly broadening its applicability [10]. ORB-SLAM2 improved upon the traditional PTAM framework by introducing a loop closure detection module and utilizing three parallel threads for tracking, local mapping, and loop closure detection, thereby laying the foundation for modern visual SLAM systems. By leveraging ORB features, ORB-SLAM2 achieved notable computational efficiency. Furthermore, the inclusion of descriptors in ORB feature points enhanced loop closure detection and relocation capabilities, particularly during wide-ranging camera motions.

In contrast to ORB-SLAM2’s feature-based approach, Forster et al. introduced Large-Scale Direct Monocular SLAM (LSD-SLAM), a direct method that bypasses feature descriptor extraction and instead computes similarity transformations directly between key frames [11]. By utilizing image regions with significant pixel gradients for bit-position tracking and depth estimation, LSD-SLAM enabled the generation of semi-dense maps, demonstrating superior performance in sparsely textured environments. Forster et al. further advanced the field with the development of Semi-Direct Visual Odometry (SVO), which combined the strengths of feature-based and direct methods [12]. Unlike traditional feature-based approaches, SVO relies on feature points only for key frame selection, eliminating the need for descriptor computation and matching, thus significantly reducing processing time. Additionally, unlike conventional direct methods, SVO does not process all pixels in each frame; instead, it uses small image patches to estimate camera motion and pose. This innovative approach not only improves real-time performance while maintaining accuracy but also incorporates three optimization techniques to ensure robust results.

Despite these advancements, V-SLAM systems often struggle with robustness in scenarios involving fast camera motion or low-light environments [13,14,15]. To address these challenges, Visual Inertial SLAM (VI-SLAM) has emerged as a powerful solution. By fusing data from visual odometers and Inertial Measurement Unit (IMU), VI-SLAM can achieve accurate 3D reconstruction of the environment and precise self-position estimation. In recent years, with the rapid development of robotics, unmanned aerial vehicles, autonomous driving, and other fields, VI-SLAM systems have been widely adopted for autonomous navigation and perception tasks [16]. For instance, Mourikis et al. enhanced robustness, speed, and accuracy by integrating IMU data with visual information under the EKF framework, enabling the system to adapt to more intense motion and texture loss [17]. Similarly, Bloesch et al. developed a monocular visual inertial odometry algorithm that demonstrated excellent tracking performance [18]. Further advancing the field, Tong Qin et al. proposed a tightly coupled approach that combines IMU measurements with feature observations to achieve highly accurate visual–inertial odometry [19]. These developments highlight the growing importance of VI-SLAM in overcoming the limitations of traditional V-SLAM systems, particularly in challenging environments.

The evolution from SLAM to VI-SLAM has marked a significant technological leap forward. While the fusion of visual and IMU data has substantially improved the robustness and precision of SLAM systems, it has not entirely overcome the intrinsic limitations of visual-based approaches. Conventional feature extraction and tracking techniques, such as optical flow, frequently encounter difficulties in environments characterized by low light, sparse textures, or dynamic elements, leading to inaccuracies in localization or system malfunctions. In contrast, deep learning methodologies have shown promise in mitigating these challenges [20,21]. This paper proposes a hybrid optical flow method that combines a Lightweight Convolutional Neural Network (LET-NET) [22] and improved Kanade-Lucas-Tomasi Tracking Method (KLT) [23] to enhance the feature extraction and tracking performance of VI-SLAM systems. In Lucas–Kanade (LK) optical flow, only two parameters of translation are used to track feature points [24], but we employ SE2 (Special Euclidean Group in 2D) to implement changes in four parameters, translation (x, y), rotation, and scale, thereby achieving better optical flow results in complex motion scenarios [25]. Although the optical flow derived from LET-NET is solely based on the network’s output images, which may lead to some data loss, our proposed hybrid optical flow method ensures high accuracy and dependable feature tracking across diverse environmental conditions. This integration aims to refine the Visual–Inertial Mapping process, offering a more reliable solution for SLAM applications.

The main contributions of this paper are as follows:

A hybrid optical flow method was proposed, which combined the improved LET-NET for robust feature extraction under dynamic lighting and blurred conditions with the enhanced tracking ability of KLT optical flow in complex motions. This hybrid method significantly improved the localization accuracy, stability, and robustness of VI-SLAM systems, especially in challenging environments with varying lighting and complex motions. The methodology underwent rigorous experimental validation utilizing publicly available datasets, demonstrating enhanced positioning precision relative to the conventional VINS-Mono approach and indicating its potential utility in practical scenarios.

## 2. Materials and Methods

### 2.1. Overview of the System

**Figure 1:**
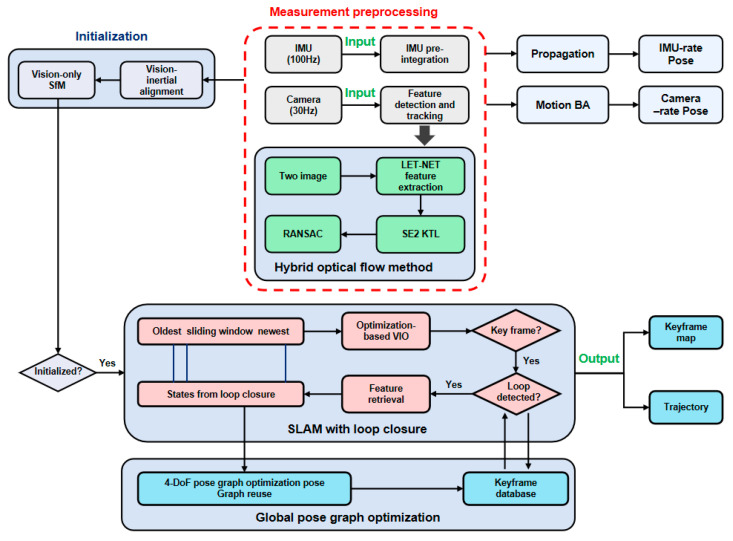
The framework of LET-SE2-VINS, comprising initialization, measurement preprocessing, hybrid optical flow, and pose graph optimization for robust SLAM.

Based on the traditional VI-SLAM framework VINS-Mono, our VI-SLAM position accuracy was improved by improving LET-NET to extract high-quality feature points and SE2 optical flow tracking, which we called LET-SE2-VINS in this research. Figure 1 shows the algorithm workflow of LET-SE2-VINS. The main components of the algorithm included deep learning-based feature extraction at the front-end, SE2 optical flow tracking at the back end, position optimization, and loop closure detection.

The IMU data were sampled at a frequency of 100 Hz, while the camera data were captured at 30 Hz. To enhance the precision of the system, the IMU data underwent pre-integration processing prior to being utilized. Both data streams were subsequently fed into the system. The system employed the LET-NET algorithm for image processing and key point extraction from the image frames. Feature points across consecutive frames were then matched using the SE2 optical flow tracking algorithm. To ensure the integrity of the matching process, the RANSAC algorithm was utilized to filter out erroneous matches. Following the front-end optimization, the system forwarded the refined feature points to the back-end processing unit. Here, key frames were established for the purpose of bitmap estimation and optimization. The feature and bitmap information associated with these key frames were then relayed to the back-end. Ultimately, the system’s global accuracy was further refined through bitmap optimization, culminating in a robust and precise localization framework.

### 2.2. Network Architecture

**Figure 2:**
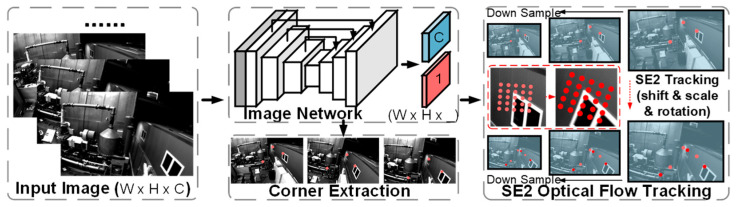
Framework of the hybrid optical flow method. The network employed a lightweight design with only four convolution operations to extract shared feature maps and generate illumination-invariant features along with key point score maps.

As shown in Figure 2, only four convolution operations were used in order to make the network design as lightweight as possible. A shared feature map of size W × H × 16 was first extracted from the input image (W × H × 3), and then a 1 × 1 convolution kernel was used to convert the shared feature map into an illumination-invariant feature map and a key point score map. Using a lightweight network, the illumination-invariant feature maps contained less high-level semantic information while retaining more low-level image information. Thus, the computation and complexity of the designed network was much lower than other networks.

Shared Encoder: The image feature encoder converted the input image I ∈ W × H × 3 into dimensions W × H × 16. The first two convolution operations used a 3 × 3 convolution kernel to extend the shared feature mapping to 8 channels. In the last layer, a 1 × 1 convolution kernel was used to increase the number of channels to 16. The ReLU [26] activation function was used after each convolution. The resolution of the original image was maintained during all convolution operations.

Feature and Score Maps Decoder: The shared feature maps were decoded into a score map and an illumination-invariant feature map by a decoding layer. The decoding layer used a 1 × 1 convolution kernel to reduce the number of channels of the feature map to 4. The first three channels were light-invariant feature maps, and the last channel was a key point score map. After convolution, the score map was activated by a sigmoid function, which restricted its value to [0, 1]. At the same time, the convolution feature map was also L2 regularized. As a result, the final output score map had a size of W × H × 1, while the size of the light-invariant feature map was W × H × 3.

For an image pair, a shallow network was used to extract the score map S and the feature map F, and a deep network was used to extract the dense descriptor map D. The shallow network here was the network structure described above. And the deep network was only used to assist in training. Key point loss, light-invariant feature loss, and descriptor loss were used to train three different outputs [S, F, D], respectively. The key point loss consisted of reprojection loss, line spike loss, and reliability loss. The NRE [27] function and mNRE function were used for descriptor loss and light-invariant feature loss, respectively.

### 2.3. SE2-Enhanced KLT Tracking

To track the motion of these points over a series of consecutive frames, we used a sparse optical flow based on the KLT. To achieve fast, accurate, and robust tracking, we also combined an inverse synthesis method described in Leutenegger’s research [28], with a similarity specification for image blocks (patches) that was not affected by intensity scaling. Several researchers suggested zero-normalized cross-correlation (ZNCC) for illumination invariant optical flow [29,30]. We used Local Sum of Squared Differences (LSSD) [31] for light-invariant optical flow, which is computationally less expensive compared to other methods.

In LK’s optical flow tracking, only two parameters of patch translation in the image were used for inter-frame tracking. However, there were also two variations of rotation and scale in the image, and we modeled these four parameters by SE2 (plane transformation matrix) to better enhance the optical flow in complex or violent movements. We formulated the patch tracking problem as estimating the transform T ∈ SE2 between two corresponding patches in two consecutive frames, which minimized the difference between the patches according to a chosen norm. Essentially, we minimized the sum of squared residuals, where each residual was defined as

(1) $$r_{i}(\xi )=\begin{array}{cc} \frac{I_{t+1}(Tx_{i})}{\bar{I_{t+1}}}-\frac{I_{t}(Tx_{i})}{\bar{I_{t}}} & \forall x_{i}\in \Omega \end{array}$$

where **I***_t_*(*x*) is the intensity of image *t* at pixel location *x*. Ω denotes the pixel block region and the mean intensity of the patch in image *t* is $\bar{I_{t}}$.

To achieve robustness to large displacements in the image, a pyramid method [32] was used in which pixel blocks were tracked, first at the tracking pixel block and then at increasingly finer levels. To weed out the wrong pixel block matches, a cross tracking method was used. The blocks of pixels were tracked from the current frame to the target frame and the points that did not return to the initial position by the second tracking were considered as outliers and discarded.

### 2.4. VINS System

The backend nonlinear optimization and closed-loop detection modules of VINS-Mono were retained. The back-end nonlinear optimization was employed to jointly optimize the camera pose by minimizing the reprojection error of visual feature points, IMU measurement residuals, and the a priori constraints on the marginalization of the sliding window; the accuracy of the optimization depends on the number of iterations, and considering the time constraints, the maximal number of iterations in this paper was set to 8. The closed-loop module carried out a loopback retrieval based on the bag-of-vision algorithm. The closed-loop was executed after being detected by the key frame database, and it was added as a closed-loop constraint item to the sliding window for local optimization. The feature points were robustly matched to determine the relative position, which was then incorporated into the sliding window as a closed-loop constraint for local optimization. Additionally, the system performed global trajectory optimization when the marginalized image from the sliding window also corresponded to a closed-loop frame.

### 2.5. Experiment

An AMD Ryzen 7 7800 × 3D CPU and NVIDIA RTX 4070 Ti Super GPU workstation, optimized for simulations. The 8-core, 96 MB 3D V-Cache CPU accelerates bundle adjustment, while the 16 GB GPU enables real-time LET-NET feature extraction (8 ms/frame). With 32 GB DDR5 RAM and a 2 TB NVMe SSD, this setup handles large-scale mapping and rapid dataset processing. The simulation time per dataset was about 2 min.

The experiment utilized the public dataset EuRoc [33] to conduct a quantitative evaluation of the entire monocular VI-SLAM system. The localization accuracy of the proposed system was compared with that of the VINS-Mono system. In this study, the localization accuracy was quantified using the Root Mean Square Error (RMSE) of the translational component from Absolute Pose Error (APE).

The EuRoc dataset employed in this experiment encompassed a variety of scenes categorized into three difficulty levels: easy, medium, and hard. Easy scenes were characterized by slow camera movements and environments with rich textures and normal lighting conditions. Medium-difficulty scenes included brief periods of darkness and camera shake. Difficult scenes presented more challenging conditions, such as significant lens shake, unclear textures, and prolonged darkness. Eleven sequences were tested in the experiment using both VINS-Mono and the method proposed in this paper. Accurate metrics were derived based on the computational results.

## 3. Results

For no-loop conditions, the localization accuracy of the algorithm proposed in this paper is superior to that of the classic VINS-Mono algorithm across all 11 sequences. In the MH_05, V1_03, and V2_03 sequences, the improvements were 27.81%, 26.33%, and 31.70%, respectively. For conditions with loop closure, the localization accuracy of the algorithm proposed in this paper is better than that of the classic VINS-Mono algorithm in 10 out of 11 sequences. In the MH_04, V1_03, and V2_03 sequences, the improvements are 35.26%, 18.83%, and 43.89%, respectively.

From the RMSE results in Table 1, it can be observed that our improved SE2-LET-VINS method achieves a lower RMSE compared to the original VINS-Mono when loop closure detection is not used (no-loop). When loop closure detection is enabled (loop), SE2-LET-VINS still performs well and achieves lower RMSE values on the MH_02, V1_01 and V2_01 datasets. From the RMSE results in Table 2, it can be seen that in more challenging EuRoc datasets, such as those with dim lighting, our proposed SE2-LET-VINS still demonstrates certain advantages, especially when loop closure detection is not used (no-loop). Compared to VINS-Mono, SE2-LET-VINS achieves lower RMSE values on most sequences. For example, on sequences with intense motion and complex scenes, such as MH_04, MH_05, and V2_03, SE2-LET-VINS-noloop reduces errors by 29.7%, 21.8%, and 24.1%, respectively, compared to VINS-Mono (no-loop). When loop closure detection is enabled (loop), SE2-LET-VINS continues to perform well, achieving lower RMSE on sequences such as MH_04, V1_02, V2_02, and V2_03. Particularly on the MH_04 sequence, the error of SE2-LET-VINS-loop is reduced by 35.3% compared to VINS-Mono (loop), indicating that the optimized optical flow tracking method can provide more consistent feature matching, thereby enhancing the global consistency of pose estimation.

Compared to SuperPoint-based systems, SE2-LET-VINS demonstrates significantly more stable and accurate pose estimation across both easy and challenging sequences of the EuRoC dataset. For example, on easy sequences like MH_01 and V2_01, SE2-LET-VINS-loop achieves RMSE reductions of 11.6% and 6.6%, respectively, relative to SuperVINS (which utilizes SuperPoint). The improvements are even more substantial on difficult sequences such as MH_04 and V2_03, with RMSE reductions of 40.6% and 5.8%, respectively. These gains underscore the advantage of SE2-LET-VINS’s SE2-based feature tracking framework combined with LETNet, which delivers superior feature consistency under challenging conditions like low light, motion blur, and texture scarcity. Consequently, this enhanced tracking robustness results in reduced drift and improved global pose consistency compared to traditional SuperPoint-based approaches.

**Figure 3:**
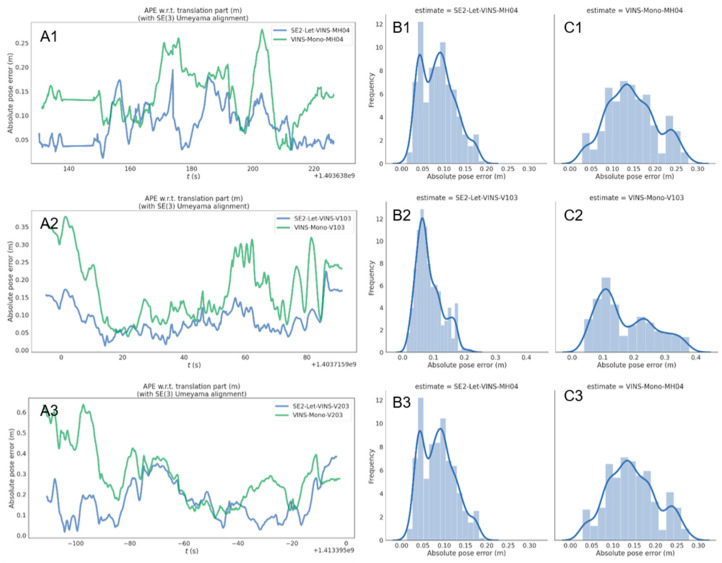
Comparison of localization accuracy between SE2-LET-VINS and VINS-Mono. (**A1**–**A3**) Error timing in the MH04 sequence, V103 sequences and V203 sequence. The green lines indicate the error distribution of the VINS-Mono and the blue lines indicate that of SE2-LET-VINS. In most cases, the values of blue lines are significantly lower than those of the green lines. (**B1**–**B3**) Gaussian fitting curve in the MH04 sequence, V103 sequences and V203 sequence using SE2-LET-VINS method. In the three tests, the probability density reached its maximum at absolute pose error (APE) value of 0.1 m, 0.07 m and 0.09 m, with corresponding values of 9.5, 12.1 and 4.8. (**C1**–**C3**) Gaussian fitting curve in the MH04 sequence, V103 sequences and V203 sequence using VINS-Mono method. In the three tests, the probability density reached its maximum at APE value of 0.13 m, 0.12 m and 0.28 m, with corresponding values of 7.1, 5.9 and 3.7.

The errors of three relatively difficult scenarios (MH04, V103, and V203) were visualized and are shown in Figure 3. There is a clear difference between SE2-LET-VINS and VINS-Mono. The blue color in the figure indicates the error distribution of the SE2-LET-VINS and the green VINS-Mono trajectories. It can be observed that the error of the SE2-LET-VINS trajectory is significantly reduced in the three difficult sequences. In addition, we performed simple statistics on the errors. It is found that the maximum, minimum, and median of the errors are greatly reduced. Therefore, it can be concluded that the improved algorithm performs significantly better than the original algorithm for localization in difficult scenarios.

The errors were fitted using a Gaussian function. From the results, it can be observed that the errors of both SE2-LET-VINS and VINS-Mono obey a Gaussian distribution. In the results, we note that the improved Gaussian fitting curve has been shifted to the left, which indicates an overall reduction in the errors. Furthermore, when looking at the Gaussian curves, we notice a difference in the error distributions. the SE2-LET-VINS has a narrower Gaussian curve, while the VINS-Mono has a wider Gaussian curve. This indicates that the stability of the error distribution has also been improved to some extent.

**Figure 4:**
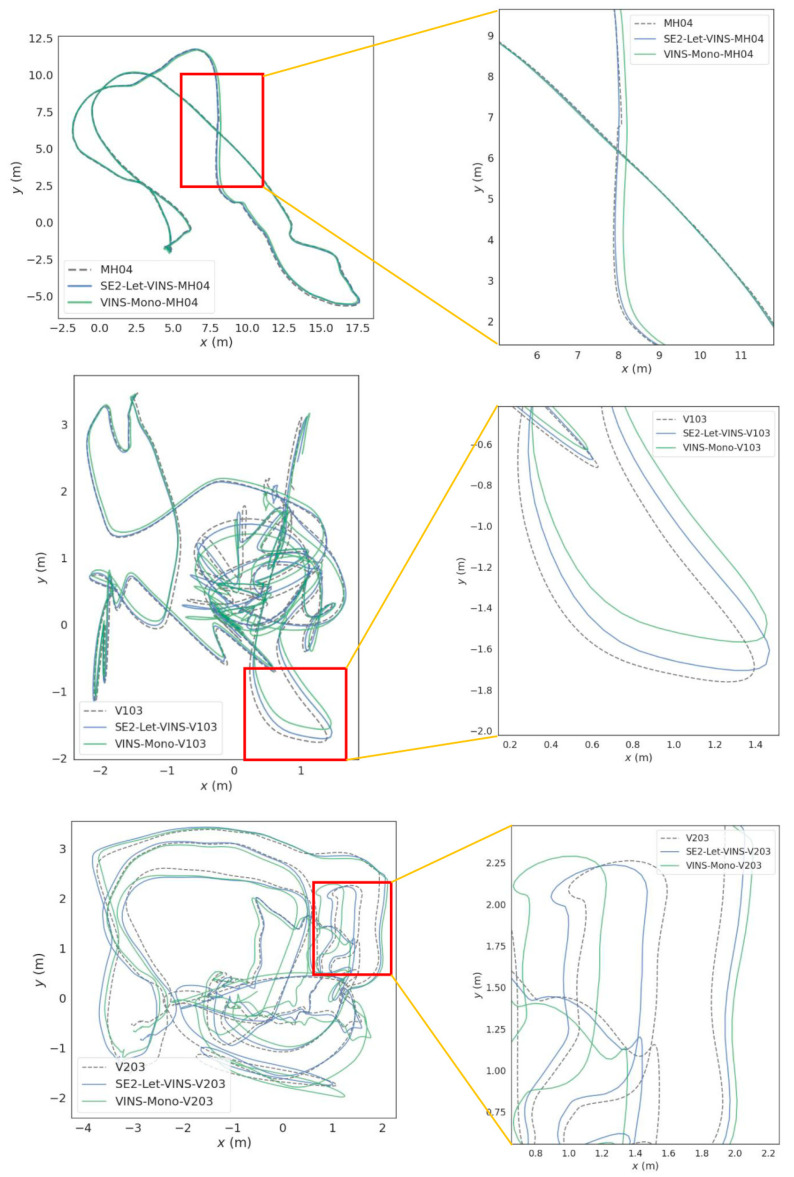
Comparison of tracking performance at three frequencies. The dot line represents the true trajectories, the blue lines represent the trajectories by our SE2-LET-VINS, and the green line represents the trajectories by VINS-Mono. The blue lines are closer to the green lines. In the MH04 sequence, SE2-LET-VINS (blue) tracks closer to the ground truth than VINS-Mono, particularly in the highlighted zoom-in region, where the VINS-Mono trajectory diverges from the true path. In the V103 sequence, the difference is more pronounced under aggressive motion. SE2-LET-VINS shows stable tracking, while VINS-Mono deviates significantly in the marked region. In V203 sequence, in scenes with high frame dropout and motion blur, SE2-LET-VINS still aligns closely with the ground truth, whereas VINS-Mono shows larger divergence in the zoom-in area.

To better illustrate the advantages of the improved algorithms in localization performance, the trajectories of both algorithms, along with the ground truth, are integrated into a single graph. The results of three sequences, MH04, V103 and V203, were selected, respectively, which represent complex scenarios and will better show the advantages and disadvantages of the localization results. In Figure 4, the left side shows the full localization trajectories, and the right side shows the localization trajectories with local zoom. The dotted lines show the true trajectories, the blue lines are the trajectories obtained using our method, and the green lines are the trajectories derived from classic VINS-Mono method. In three experiments under different frequencies, our trajectories consistently demonstrate a closer approximation to the true trajectories, showcasing superior tracking performance.

**Figure 5:**
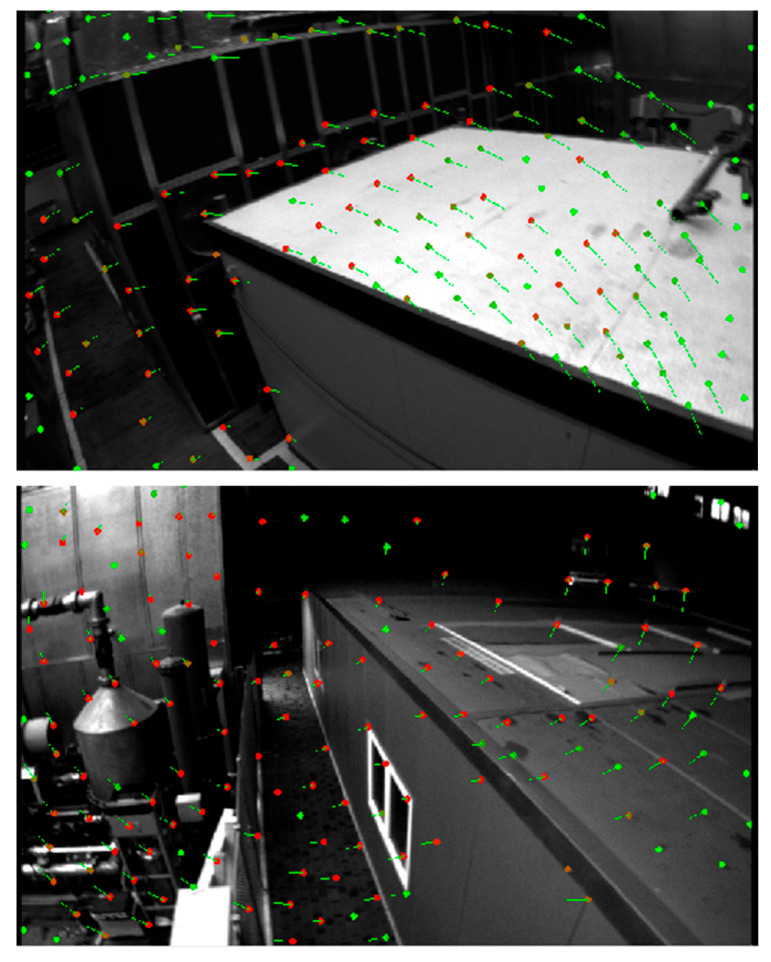

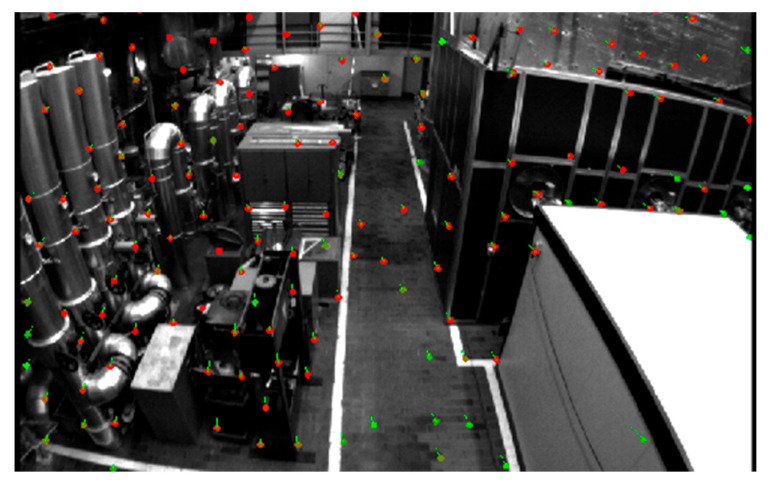
Results of feature extraction and tracking on the MH_03 dataset. The green points represent detected feature points in the current frame, while the red points indicate the corresponding matched points in the next frame. The arrows show the optical flow displacement vectors. It can be observed that our method maintains stable and accurate feature tracking even under varying perspectives, illumination conditions, and in areas with weak textures or repetitive structures, demonstrating strong robustness in challenging real-world environments.

**Figure 6:**
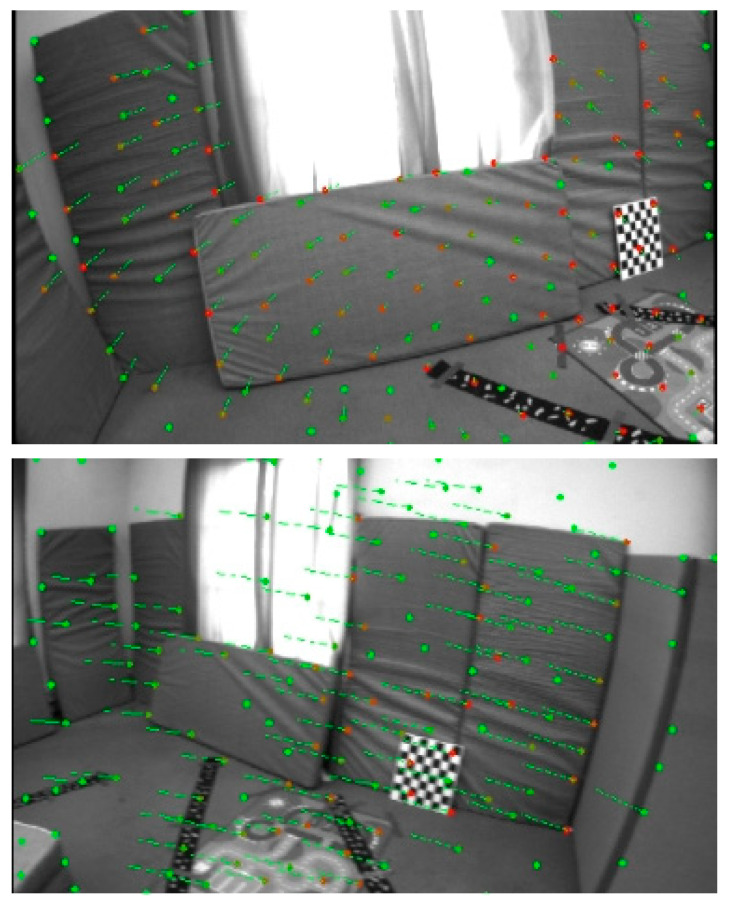

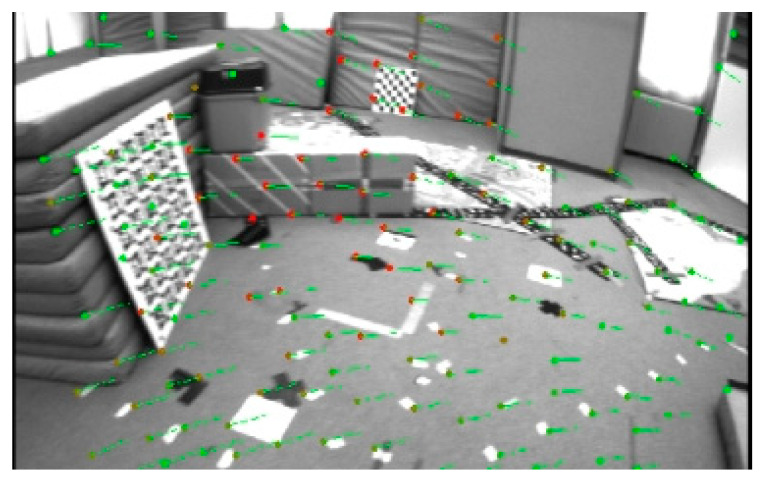
Results of feature extraction and tracking on the V1_02 dataset. Green points denote the detected feature points in the current frame, and red points indicate the corresponding matches in the next frame. Despite the presence of strong lighting from the windows, repetitive patterns, and soft-textured surfaces like cushions, the proposed SE2-LET-VINS method consistently maintains dense and stable feature tracking. This demonstrates the method’s robustness to illumination changes and low-texture environments.

**Figure 7:**
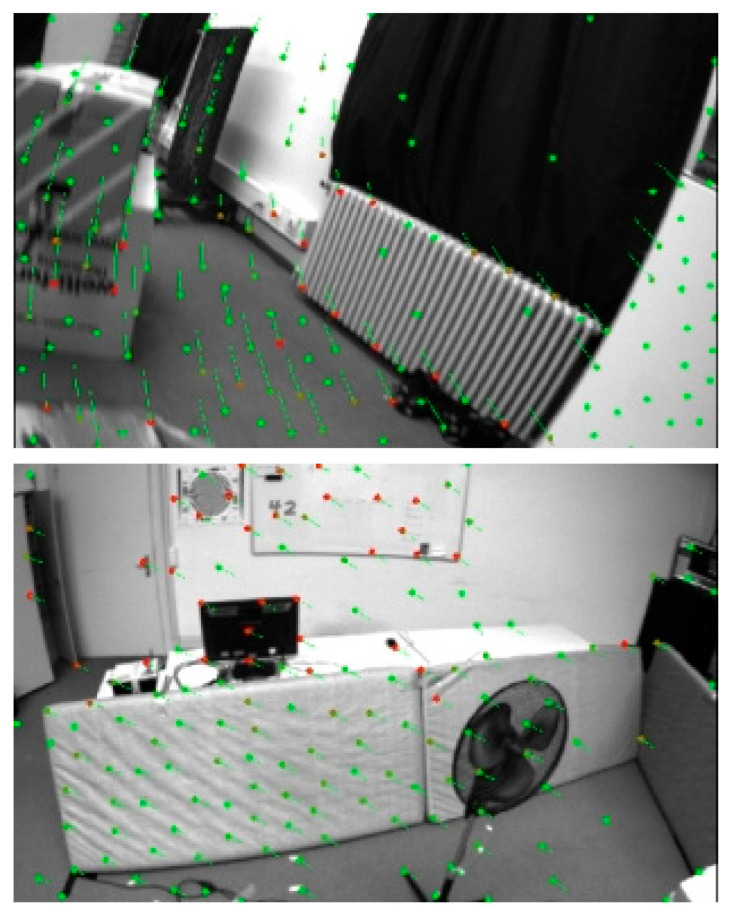

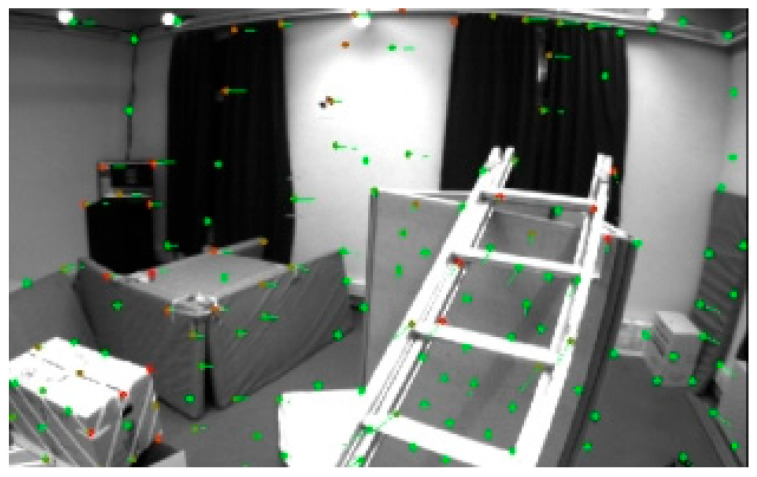
Results of feature extraction and tracking on the V2_02 dataset. The green dots represent extracted features in the current frame, and the red dots show their tracked positions in the next frame. This indoor scene contains weak textures, motion blur, and occlusions (e.g., ladders and curtains). Despite these challenges, our method achieves consistent and reliable feature tracking, highlighting its robustness in low-texture and partially occluded environments.

As shown in Figure 5, Figure 6 and Figure 7, the proposed SE2-LET-VINS method demonstrates stable and consistent feature tracking across diverse scenes in the MH_03, V1_02, and V2_02 datasets. Despite significant viewpoint changes, lighting variations, motion blur, and the presence of texture-less or repetitive surfaces, the algorithm consistently maintains accurate correspondences between consecutive frames without notable feature loss or drift. The green and red arrows indicate successfully matched feature points, illustrating the system’s ability to preserve feature integrity and temporal consistency throughout the sequence.

Notably, in environments with strong lighting contrast (e.g., window glare in V1_02) and sparse features (e.g., smooth walls and floors in V2_02), our method still achieves reliable tracking, where traditional methods often struggle. This confirms the effectiveness of the hybrid optical flow formulation, which integrates deep-learned features from LET-NET with SE2-based geometric constraints, enabling both robustness and computational efficiency.

Moreover, the method’s resilience to partial occlusions and abrupt perspective changes (such as ladder obstructions or narrow corridors) further highlights its practicality for real-world applications. These results validate that the proposed system is not only theoretically sound but also highly adaptable to varied and complex visual environments, making it well-suited for long-term SLAM and autonomous navigation tasks.

## 4. Discussion

The SE2-LET-VINS algorithm proposed in this paper demonstrates significant performance improvements across multiple test sequences, particularly in complex scenarios. Compared to the classic VINS-Mono algorithm, our method shows notable enhancements in both localization accuracy and robustness. Under the no-loop conditions, the accuracy of our algorithm improves by 27.81%, 26.33%, and 31.70% for the challenging sequences MH_05, V1_03, and V2_03, respectively. In the presence of loop closure detection, the accuracy in the MH_04, V1_03, and V2_03 sequences increased by 35.26%, 18.83%, and 43.89%, respectively. These results highlight the significant advantages of SE2-LET-VINS in handling complex and dynamic environments. Furthermore, the trajectory visualization results further validate the practical advantages of our algorithm. For instance, in the MH_04, V1_03, and V2_03 sequences, the trajectories produced by SE2-LET-VINS show better alignment with the ground truth, particularly in the zoomed-in views, where it is evident that our algorithm is better able to track the true trajectory in complex environments with dynamic lighting changes, significantly reducing error accumulation. To further verify the robustness of our feature tracking approach, we conducted detailed qualitative experiments using representative sequences from the MH_03, V1_02, and V2_02 datasets. These results show that SE2-LET-VINS maintains stable and consistent feature tracking across diverse scenes, even under significant viewpoint changes, low-texture regions, motion blur, and varying illumination.

In summary, SE2-LET-VINS offers clear advantages over the traditional VINS-Mono algorithm in terms of both localization accuracy and robustness. By combining SE2 optical flow tracking with LET-NET feature extraction, our algorithm demonstrates higher stability and accuracy when dealing with challenging localization tasks in complex environments, particularly under conditions such as low lighting and rapid motion.

## 5. Conclusions

The SE2-LET-VINS algorithm proposed in this paper demonstrates significant advancements over the classic VINS-Mono algorithm in terms of localization accuracy, robustness, and tracking performance, particularly in complex and challenging scenarios. Under no-loop conditions, our algorithm achieves substantial improvements in localization accuracy, with enhancements of 27.81%, 26.33%, and 31.70% for the MH_05, V1_03, and V2_03 sequences, respectively. When loop closure detection is enabled, SE2-LET-VINS further outperforms VINS-Mono, with accuracy improvements of 35.26%, 18.83%, and 43.89% for the MH_04, V1_03, and V2_03 sequences, respectively. These results underscore the effectiveness of our approach in handling dynamic and challenging environments.

The RMSE results from both easy and hard EuRoc datasets confirm that SE2-LET-VINS consistently achieves lower errors compared to VINS-Mono, particularly in scenarios with dim lighting, rapid motion, and complex scenes. For instance, in the MH_04, MH_05, and V2_03 sequences, SE2-LET-VINS reduces errors by 29.7%, 21.8%, and 24.1%, respectively, under no-loop conditions. Even with loop closure detection enabled, SE2-LET-VINS maintains superior performance, with significant error reductions in sequences such as MH_04, V1_02, V2_02, and V2_03.

Visualization of the trajectories further validates the practical advantages of SE2-LET-VINS. In complex scenarios like MH_04, V1_03, and V2_03, the trajectories generated by our algorithm align more closely with the ground truth, demonstrating better tracking performance and reduced error accumulation. The Gaussian fitting analysis also reveals that SE2-LET-VINS exhibits a narrower error distribution, indicating improved stability and consistency in localization.

In addition to quantitative evaluation, qualitative visualizations of feature tracking and trajectory alignment further highlight the practical advantages of SE2-LET-VINS. As shown in Figure 5, Figure 6 and Figure 7, the system demonstrates stable and consistent feature tracking across diverse environments, including industrial, indoor, and cluttered scenes. Despite significant viewpoint changes, lighting variations, and texture-less surfaces, the algorithm maintains accurate feature correspondences between frames, without substantial loss or drift. The effective integration of LET-NET-based deep feature extraction and SE2-based optical flow enables robust tracking even under occlusions, motion blur, and reflective surfaces.

In conclusion, the SE2-LET-VINS algorithm, which integrates SE2 optical flow tracking with LET-NET feature extraction, offers a robust and accurate solution for visual-inertial navigation systems. It excels in challenging environments characterized by low lighting, rapid motion, and complex scenes, making it a significant improvement over the traditional VINS-Mono algorithm. Future work will focus on further optimizing the algorithm for real-time applications and extending its capabilities to even more diverse and demanding scenarios.

## Figures and Tables

**Table 1:** The RMSE results on the easy EuRoc dataset.

Sequences	VINS-Mono-Noloop	SE2-LET-VINS-Noloop	VINS-Mono-Loop	SE2-LET-VINS-Loop	SuperVINS
MH_01	0.191328	0.1593398	0.090035	0.0969550	0.086656
MH_02	0.167241	0.1445446	0.077075	0.0543076	0.096954
V1_01	0.078908	0.0776242	0.046719	0.0428872	0.201021
V2_01	0.085285	0.0846368	0.067454	0.0572118	0.061106

**Table 2:** The RMSE results on the hard EuRoc dataset.

Sequences	VINS-Mono-Noloop	SE2-LET-VINS-Noloop	VINS-Mono-Loop	SE2-LET-VINS-Loop	SuperVINS
MH_03	0.217923	0.2112980	0.072412	0.0687790	0.180578
MH_04	0.377912	0.2658094	0.156727	0.1014580	0.170761
MH_05	0.330936	0.2589238	0.145881	0.1422262	0.158304
V1_02	0.097653	0.0862660	0.071733	0.0554892	0.115990
V1_03	0.147090	0.1164328	0.13369	0.1085114	0.221410
V2_02	0.121924	0.1189912	0.108744	0.0756444	0.100303
V2_03	0.323834	0.2458836	0.318238	0.1785496	0.168746

## Data Availability

The data used in this research are available upon request.
